# SARS-CoV-2 evolution during prolonged infection in immunocompromised patients

**DOI:** 10.1128/mbio.00110-24

**Published:** 2024-02-16

**Authors:** Andrew D. Marques, Jevon Graham-Wooten, Ayannah S. Fitzgerald, Ashley Sobel Leonard, Emma J. Cook, John K. Everett, Kyle G. Rodino, Louise H. Moncla, Brendan J. Kelly, Ronald G. Collman, Frederic D. Bushman

**Affiliations:** 1 Perelman School of Medicine, University of Pennsylvania, Philadelphia, Pennsylvania, USA; 2 Division of Pulmonary, Allergy, and Critical Care, Philadelphia, Pennsylvania, USA; 3 Division of Infectious Diseases, Children’s Hospital of Philadelphia, University of Pennsylvania, Philadelphia, Pennsylvania, USA; 4 Department of Pathobiology, University of Pennsylvania, Philadelphia, Pennsylvania, USA; Washington University in St Louis School of Medicine, St. Louis, Missouri, USA

**Keywords:** COVID-19, SARS-CoV-2, coronavirus, long-term infection, prolonged infection

## Abstract

Prolonged infection with severe acute respiratory syndrome coronavirus 2 (SARS-CoV-2) in immunocompromised patients provides an opportunity for viral evolution, potentially leading to the generation of new pathogenic variants. To investigate the pathways of viral evolution, we carried out a study on five patients experiencing prolonged SARS-CoV-2 infection (quantitative polymerase chain reaction-positive for 79–203 days) who were immunocompromised due to treatment for lymphoma or solid organ transplantation. For each timepoint analyzed, we generated at least two independent viral genome sequences to assess the heterogeneity and control for sequencing error. Four of the five patients likely had prolonged infection; the fifth apparently experienced a reinfection. The rates of accumulation of substitutions in the viral genome per day were higher in hospitalized patients with prolonged infection than those estimated for the community background. The *spike* coding region accumulated a significantly greater number of unique mutations than other viral coding regions, and the mutation density was higher. Two patients were treated with monoclonal antibodies (bebtelovimab and sotrovimab); by the next sampled timepoint, each virus population showed substitutions associated with monoclonal antibody resistance as the dominant forms (*spike* K444N and *spike* E340D). All patients received remdesivir, but remdesivir-resistant substitutions were not detected. These data thus help elucidate the trends of emergence, evolution, and selection of mutational variants within long-term infected immunocompromised individuals.

**IMPORTANCE:**

SARS-CoV-2 is responsible for a global pandemic, driven in part by the emergence of new viral variants. Where do these new variants come from? One model is that long-term viral persistence in infected individuals allows for viral evolution in response to host pressures, resulting in viruses more likely to replicate efficiently in humans. In this study, we characterize replication in several hospitalized and long-term infected individuals, documenting efficient pathways of viral evolution.

## INTRODUCTION

The severe acute respiratory syndrome coronavirus 2 (SARS-CoV-2) pandemic has been characterized by the regular emergence of new variants. The origin of these new variants is unclear, with some speculating emergence from zoonotic spillover into other vertebrates and spill back into humans. (1–5). An alternative and widely discussed potential source of new variants is infection in immunocompromised patients—in these patients, the virus is resident long term and so can be exposed to a series of selective pressures in a weakened host that may develop sub-optimal immune responses, allowing changes to accumulate in response in the viral genome (4, 6, 7).

Several studies have examined the viral genetic changes that occur during infection in immunocompromised individuals, providing compelling evidence that they are linked to unusually high rates of within-host mutation accumulation (6–26). These studies have monitored individuals with various immunocompromising conditions, such as organ transplant patients on immunosuppressive drug treatment, cancer patients undergoing chemotherapy or immunotherapy, patients on immunosuppressive drug treatment for autoimmune disorders, and people with HIV infection. Previous studies have characterized at least 43 human patients and identified multiple examples of immune escape and drug-resistant amino acid substitutions (Table S1). In one study, 13.9% of B cell lymphoma patients infected with SARS-CoV-2 had infections lasting 30 days or greater (27). Some individuals with B cell lymphoma have reduced ability to produce SARS-CoV-2-neutralizing antibodies, placing them at higher risk for prolonged infection (28–30) and failure to respond to vaccination (31, 32). In one example, an individual with B cell lymphoma sustained a SARS-CoV-2 infection for 156 days, with the virus accumulating 16 mutations that included four neutralizing antibody escape substitutions (19). In another example, a group of immunocompromised heart transplant patients independently developed the E484K immune evasion substitution in spike within just 14 days (12). The main driver of selection in many of these cases is thought to result from selective pressure for increased fitness of cell–cell transmission within the host (33). These studies suggest that new SARS-CoV-2 variants may accumulate rapidly in immune-compromised individuals, though this has not been extensively quantified.

In this study, we performed genomic analysis of five immunocompromised patients with suspected prolonged SARS-CoV-2 infections to monitor the emergence of new intrahost single-nucleotide variants (iSNVs). To minimize the influence of sequencing error and account for within-host heterogeneity, we generated at least two independent viral whole-genome sequences per timepoint, allowing us to focus on iSNVs that are reproducibly detected. Using these data, we characterize viral evolutionary dynamics within hosts, monitor the development of drug resistance, and compare evolution rates to those seen in the nonimmunocompromised population.

## RESULTS

### Genomic signatures of prolonged infection

**Fig 1:**
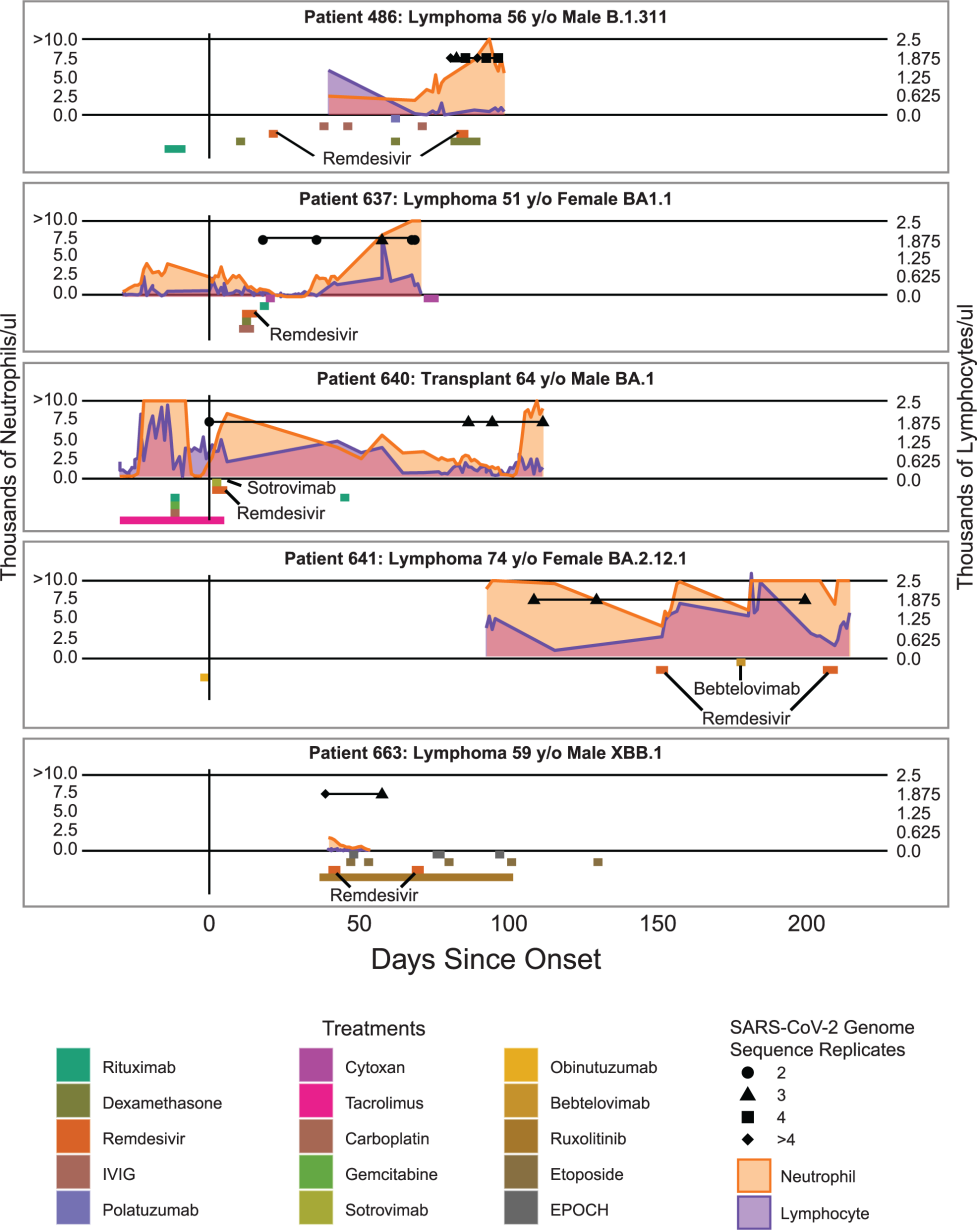
Timeline showing days since symptom onset along the x-axis and patient data on the y-axis. Treatments are colored as boxes below the x-axis, where the length of the box represents the window of treatment administration. Orange and purple line graphs represent absolute neutrophil count (ANC) and absolute lymphocyte count (ALC), respectively, across the left and right y-axis with units of thousands of cells per microliter. Black shapes represent replicates for a given timepoint, and the horizontal black line indicates the period for which sequence data are available. The black vertical line represents the day of onset either by symptom if applicable or by first positive test if initially asymptomatic.

**Fig 2:**
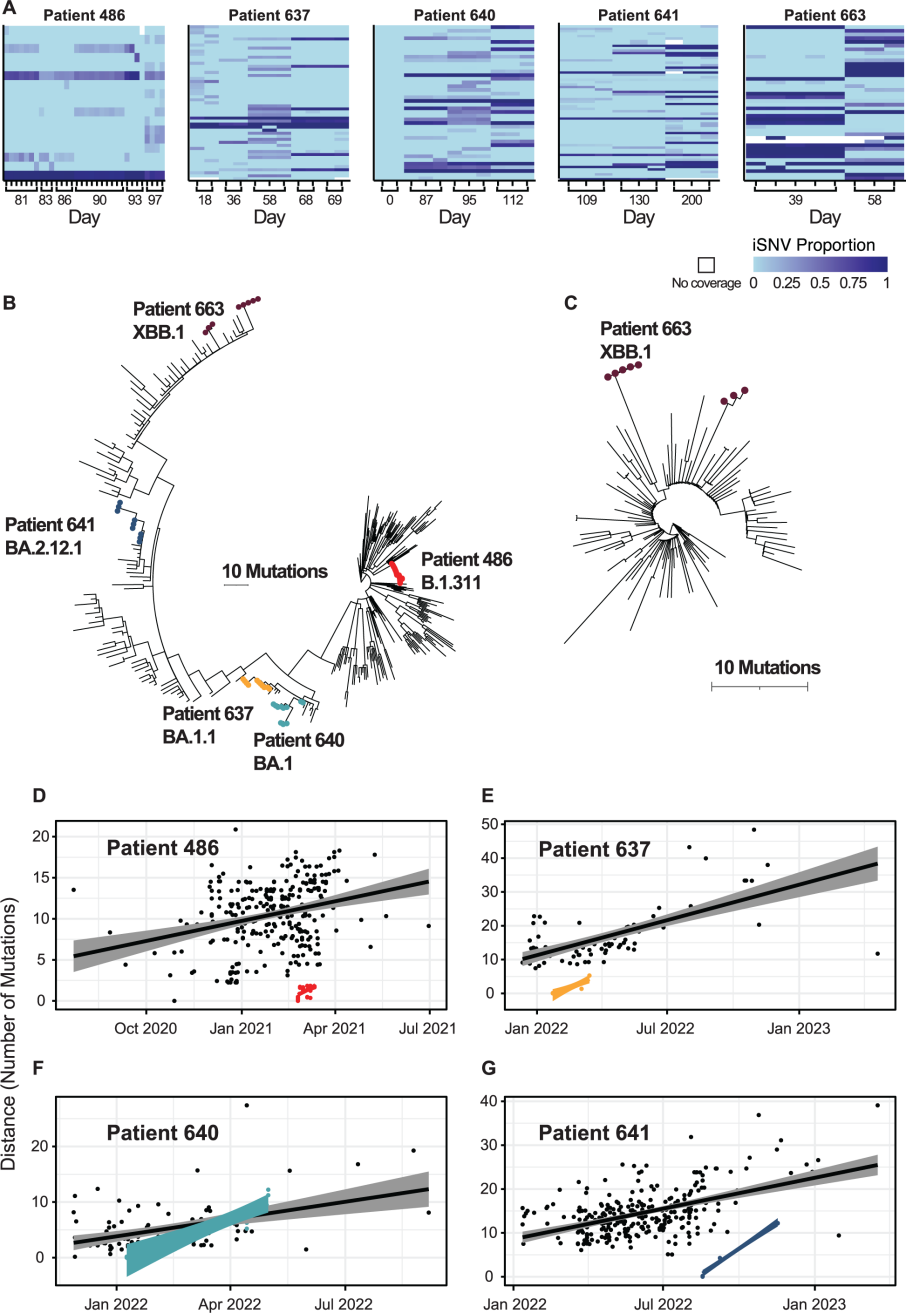
Overview of SARS-CoV-2 evolution in long-term infected individuals. (A) Heatmap showing timepoints and replicates for each patient. Columns represent the genome sequence samples, and rows represent mutations, where light blue indicates a match to the ancestral Wuhan reference strain, and the darker shades indicate increasing prevalence. Only mutations that were found in more than one replicate with >0.03 occurrence and showed changes over the course of infection are included in the heatmap. (B) Phylogenetic tree showing patients in a representative tree covering the course of the pandemic. (C) Phylogenetic tree of patient 663, the case of suspected reinfection, shown in the context of subsampled sequences representing viral lineage XBB.1. (D) Root-to-tip plot for infection in patient 486 (red) and background (black) of the same viral strain, B.1.311. (E) Root-to-tip plot for infection in patient 637 (orange) and background (black) of the same viral strain, BA.1.1. (F) Root-to-tip plot for infection in patient 640 (light blue) and background (black) of the same viral strain, BA.1. (G) Root-to-tip plot for infection in patient 641 (dark blue) and background (black) of the same viral strain, XBB.1.

We enrolled 27 immunocompromised patients persistently infected with SARS-CoV-2 (>21 days positive by the nucleic acid amplification test). Of these, five patients had samples spanning multiple days of collection where viral whole-genome sequencing returned high-quality genomes (Fig. 1). We defined high-quality genomes as having >200 × mean coverage with no more than 2% ambiguous calls. A total of 71 replicates representing 20 unique samples were recovered, with 64 of 71 replicates having a mean coverage >1,000 × (Fig. 2A; Table S2). We then selected samples with at least two independent replicates of the viral genome sequence per timepoint to ensure reproducibility. Only patients with at least two replicates from at least two timepoints were included. Replicates at each timepoint were largely consistent in the mutations identified and the proportional occurrence of each of these mutations (Fig. S1). We recovered data from patients 486, 637, 640, 641, and 663, who had prolonged infections, as observed by quantitative polymerase chain reaction (qPCR)-positive tests, lasting 97, 86, 112, 203, and 79 days, respectively (Fig. S2). From these, viral genomic data were recovered spanning 16, 51, 112, 91, and 19 days of infection (Table S3). Patients 486, 637, 641, and 663 were immunocompromised patients with B cell lymphoma; patient 640 was a heart transplant recipient (Table S3 and S4).

### Identifying an example of suspected reinfection

The five patients tested positive by reverse transcription-quantitative polymerase chain reaction (RT-qPCR) for viral RNA over time, but it was possible that the initially infecting strain might have been replaced by a different newly infecting strain over the sampling period. We thus generated phylogenetic trees containing genomes for each of the patients together with genomes from the community background using IQ-Tree and Nextclade (34–36). To characterize the community background, we subsampled genomes from the same lineage found within the United States and with 75% of isolates from our geographical region (Pennsylvania, New Jersey, and Delaware); the remaining 25% of background isolates came from other locations within the United States. Following alignments, IQ-Tree was used to infer maximum-likelihood trees using 1,000 bootstrap replicates (37). These trees contextualize the prolonged infection samples within representative sampling of contemporaneous, genetically similar surveillance isolates. We found that one patient, 663, had two distinct clusters separated by contemporaneous, genetically similar background sequences. Co-infection is not likely, as indicated by a lack of shared iSNVs from the earliest timepoint and later timepoint (Fig. 2A; Table S6); however, super-infection at an intermediate timepoint cannot be ruled out. The discrete phylogenetic clusters of 663 isolates suggest that the two timepoints represented distinct strains and thus a likely reinfection with potential super-infection (Fig. 2B and C; Fig. S3).

### Accelerated within-host viral evolution rates

The remaining four patients had all timepoint/replicates clustered together, consistent with prolonged infections caused by a single strain (Fig. S3). Fig. 2B places the prolonged-infection patients in the context of the global population evolution beginning with the emergence of SARS-CoV-2 in late 2019. We observed an accumulation of up to 18 consensus mutations in the patient sampled over the longest period (patient 640, sampled over 112 days).

To estimate and compare evolutionary rates, we calculated the root-to-tip distances from the patient-specific phylogenetic trees with contemporaneous, genetically similar subsampled background isolates (Fig. 2D through G; Fig. S3). Each tree was rooted with the earliest detected isolate of that lineage in the United States with a complete genome submitted to GISAID. The root for the prolonged-infection individual was assigned to the earliest sequence of the highest mean coverage that was recovered; therefore, the pairwise distance to subsequent timepoints represents the genetic distance from the earliest known point. Evolutionary rate is defined in this study as the number of consensus mutations that accumulated from the root to tip per year. This allows for direct comparison of rates of mutation accumulation within a single host versus individuals sampled across the regional population. To estimate the rate of mutation accumulation in each prolonged infection, we modeled the number of consensus mutations that accumulated in the genome as a function of time (in months) with linear regression. For patients with fewer than 2 months of sequence data, the evolution rate of the prolonged-infection patients was higher than background rates but did not achieve significance. The evolution rate for patient 486 was 1.47 consensus mutations per month (95% confidence interval [95% CI], 0–3.12) with the background evolution rate of 0.82 (95% CI, 0.52–1.12), and the evolution rate for patient 637 was 2.82 (95% CI, 1.48–4.16) with the background evolution rate as 1.75 (95% CI, 1.39–2.12). For patients with more than 2 months of sequence data, we observed statistically greater evolution rates than background. The evolution rate for patient 640 was measured as 2.78 consensus mutations per month (95% CI, 1.69–3.87) with the background evolution rate as 0.89 (95% CI, 0.41–1.37), and the evolution rate for patient 641 was 3.83 (95% CI, 3.17–4.5) and the background evolution rate was 1.33 (95% CI, 1.1–1.56). Together, these data suggest that the measured evolution rates of prolonged-infection immunocompromised individuals were 1.6–3.1 times higher than those of contemporaneous samples of a similar genetic background. For sampling windows that were longer than 2 months in duration, we measured a greater evolutionary rate in immunocompromised prolonged infections than expected compared with the community background, which is consistent with the finding of a previous report (38).

### Accumulation and persistence of minor variants

**Fig 3:**
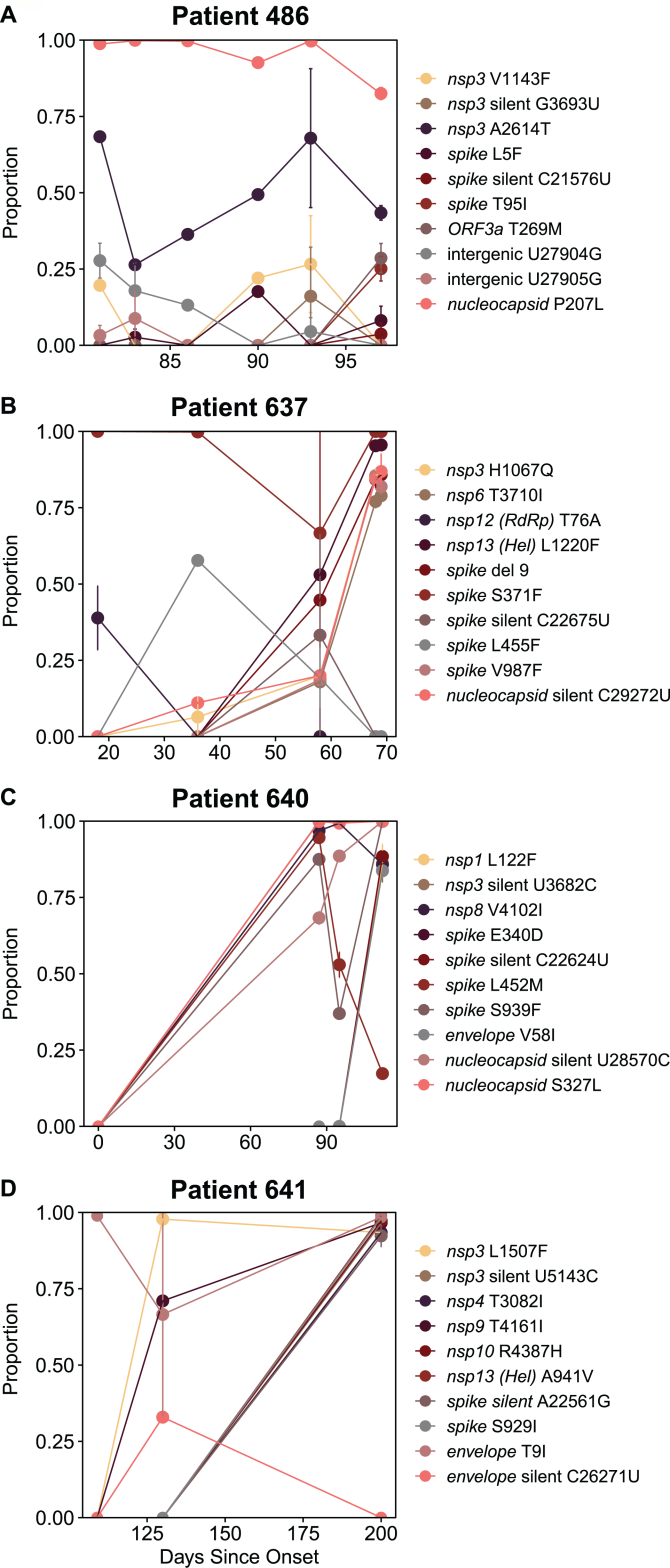
Summary of the top 10 most variable iSNVs within each patient. Different mutations are distinguished by color. Error bars show variability across replicates; lack of error bars indicates less than 0.001 deviation in proportion. (A) Patient 486’s top 10 most variable iSNVs. (B) Patient 637’s top 10 most variable iSNVs. (C) Patient 640’s top 10 most variable iSNVs. (D) Patient 641’s top 10 most variable iSNVs.

To investigate changes in viral sequences in more detail, we examined the emergence and changing proportions of intrahost single-nucleotide variants (iSNV) over the sampling period. Fig. 3A through D displays the 10 most rapidly changing iSNVs for each of the patients. For patient 640, data were available for the earliest stage of infection. This individual was initially asymptomatic when first tested positive, with subsequent symptom onset; therefore, day 0 represents the first positive test. Unique to our data set, this is the only timepoint with no iSNVs present in our data set, suggesting the dominance of a single viral strain (Fig. 3C). The absence of iSNVs at this patient’s timepoint is consistent with previous findings of tight bottlenecks at transmission (39). At later timepoints for this patient, and for all other patients’ timepoints, iSNVs are detectable across replicates.

**Fig 4:**
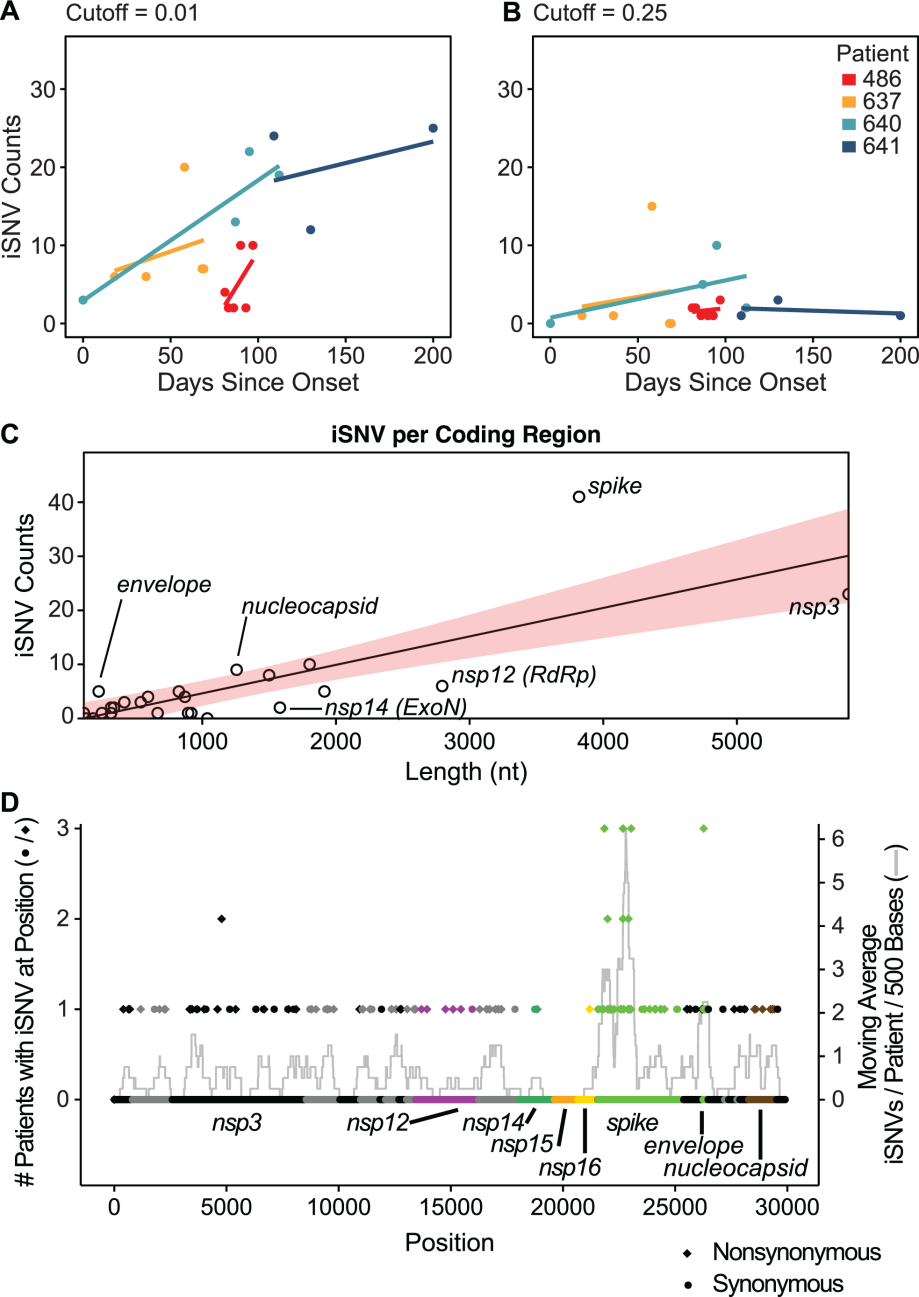
iSNV accumulation over time and by the coding region. (A) iSNV accumulation across patients, shown over the full duration of sampled infection. Linear regression lines were fitted for a cutoff of 0.01 (iSNV found between 1% and 99% prevalence). (B) iSNV accumulation for a cutoff of 0.25 (25%–75% prevalence). (C) Number of iSNVs by the coding region showing length as a nucleotide on the x-axis and iSNV counts on the y-axis. Shaded region indicates the 95% CI for linear regression of coding region length to iSNV counts. (D) Occurrence of iSNVs throughout the genome by position. The left axis indicates the number of patients with any iSNV in a given position, indicated by either circles or diamonds. Circles indicate that the iSNV was synonymous, and diamonds indicate that the iSNV was nonsynonymous. The right axis indicates the moving average centered on each position spanning a window of 500 bases. Units for the moving average are average iSNVs per patient per 500 bases. Coding regions are colored in alternating black or gray, with the regions of interest labeled and colored.

We hypothesized that over the course of infection, there would be an increased accumulation of iSNVs. For the remainder of the study, we classify iSNVs as a mutation within the cutoff range detected in two or more replicates at a single timepoint. For example, a cutoff value of 0.01 would account for iSNVs that are between 0.01 and 0.99 of reads for that position. Not surprisingly, we found that at lower thresholds, there was an increasing accumulation of iSNV compared to higher thresholds, which are largely unchanged over the duration of infection (Fig. 4A and B; Fig. S4). At a cutoff of 0.01, we found the accumulation of iSNVs at a rate of 10.9 (95% CI, −4.8 to 26.6), 2.3 (95% CI, −6.6 to 11.2), 4.6 (95% CI, 1.9 to 7.3), and 1.6 (95% CI, −6.7 to 10.0) SNVs per month for patients 486, 637, 640, and 641. At this threshold, an average of 4.9 iSNVs accumulated with each month of infection. Relatively low abundance iSNVs increased in frequency with time, while the frequency of more abundant iSNVs remained more constant.

### iSNV mutation density within the SARS-CoV-2 genome

To estimate whether iSNVs accumulate preferentially in specific coding regions compared to others, we aggregated iSNVs by the coding region and normalized based on coding region size. The iSNV counts per coding region are depicted in Fig. 4C. We found that coding regions with the fewest detected iSNVs include *nsp12* (RNA-dependent RNA-polymerase), *nsp14* (exonuclease), and *nsp2* (reprograms host translation machinery) (40; Fig. 4D). These coding regions are also found to be less variable over the course of the global pandemic (41–43). The coding regions with the highest iSNV counts were *spike*, *nucleocapsid*, and *envelope. Spike* was the only coding region with a statistically significant greater accumulation of iSNVs compared to other coding regions after using the Benjamini–Hochberg procedure to control for the false discovery rate (adj. *P* = 0.014). Across the four patients, we identified 41 unique *spike* iSNVs (Fig. 4D). Moreover, we found that positions with iSNVs across more than one patient were consistently nonsynonymous substitutions (Fig. 4D). Thus, in immunocompromised hosts, we observe preferential accumulation of substitutions in *spike* relative to the rest of the genome, as has been observed in the general population (44).

### Within-host evolution and selection

**Fig 5:**
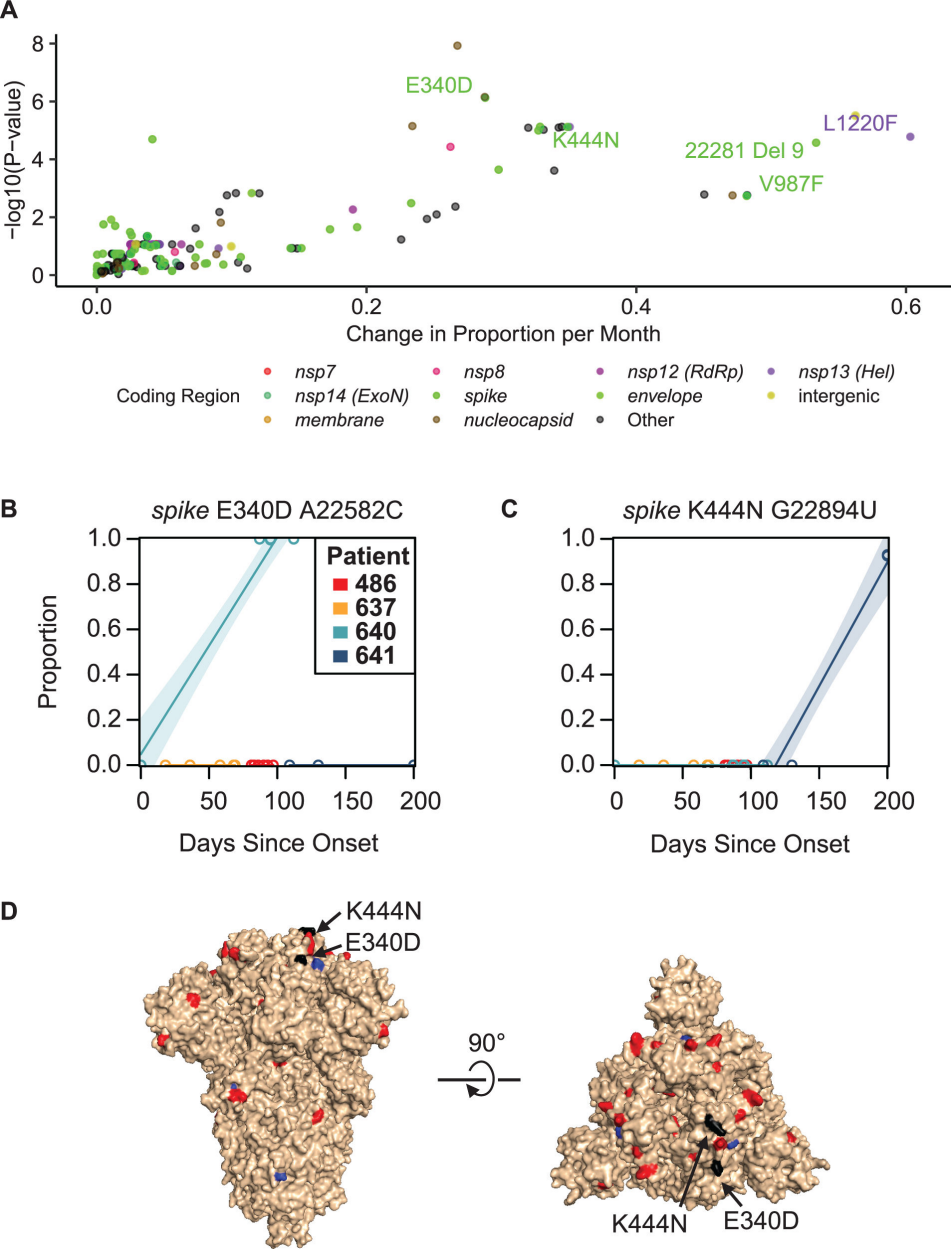
Rate of change of mutations and evasion of monoclonal antibody therapy. (A) Plot showing the change in proportion per month for iSNVs and *P*-values indicating a certainty that there was a change in iSNV proportion, colored by the coding region. (B) Plot showing *spike* E340D, which confers sotrovimab resistance, across all patients. (C) Plot showing *spike* K444N substitution, which confers resistance to bebtelovimab, across all patients. (D) Structure of SARS-CoV-2 *spike* showing nonsynonymous iSNVs as red, synonymous iSNVs as blue, and known drug-resistant mutations that rose to dominate in our cohort marked in black.

To assess the evolution and selection within patients, we used linear regression to estimate the rate of iSNV change in proportion over time within each infected patient for all iSNVs that appear in multiple replicates with >3% occurrence (Fig. S5). This threshold was selected because it was effective at removing false-positive iSNVs that were not reproducible in replicate sequencing (39, 45, 46). Slopes provided estimates of rates of change in iSNV frequency per month (Fig. 5A).

We identified several mutations in our patients, which were concordant with published data on long-term infected patients. Helicase L1220 is a position that rarely experiences mutations in random surveillance samples (<0.1% of uploaded sequences, 10,414/16,351,920, GISAID data accessed 12/20/2023) but has previously been identified as having mutations including L1220I in lymphoma patients with depleted B cells; here, we identified L1220F in patient 637 (47–50). *Spike* V987F emerged in patient 637, which was reported previously in a B cell lymphoma patient with prolonged infection but is rarely observed in surveillance samples (<0.01% of uploaded sequences, 1,084/16,351,920, GISAID data accessed 12/20/2023) (48–51). Kemp et al. identified a decrease in *spike* T240I in a prolonged-infecton individual; we identified a deletion of nine nucleotides in this region (starting at position 22281), suggesting another common site found in immunocompromised individuals (15). We speculate that the presence of these mutations in prolonged infections could be explained by a replication fitness advantage in immunocompromised subjects, while in immunocompetent subjects, these mutations allow immune recognition, which constrains their emergence.

### Antibody and drug escape mutations

We hypothesized that viruses replicating in patients treated with antiviral drugs or monoclonal antibodies (mAbs) would develop mutations conferring drug resistance. Two of the patients received mAb treatments (bebtelovimab or sotrovimab), which target the SARS-CoV-2 spike protein to neutralize the virus (37, 39). In both cases, mAb-resistant mutations replaced the dominant amino acid by the next timepoint following antibody administration (Fig. 5B through D). The bebtelovimab-resistant mutation, *spike* K444N, increased from a prevalence of 0% to 92.7% within 28 days post-treatment. The sotrovimab-resistant mutant *spike* E340D went from 0% to 99.9% prevalence within 87 days post-treatment. These findings are consistent with previous reports where mAbs were administered and resistance was detected (7, 9, 24).

In contrast to mAb treatment, the drug remdesivir was administered to all of the patients studied here, but known remdesivir-resistant mutations were not detected (52, 53).

## DISCUSSION

Understanding evolutionary trajectories in SARS-CoV-2 is critical for pandemic preparedness and optimizing therapeutic strategies. Surveillance in immunocompromised individuals with prolonged infections allows for a greater understanding of potential sources of new variants that may emerge and circulate in the wider population. Treatment regimens used in these individuals may result in accumulation of new drug-resistant substitutions that could result in less effective future treatments after transmission to the broader community. In this study, we performed genomic analysis of five potential SARS-CoV-2 prolonged infections, finding four of five to be likely true prolonged infections—the fifth was a likely reinfection. For inclusion, we required recovery of at least two independent viral whole-genome sequences from at least two timepoints, allowing for reproducible detection of novel sequence variants.

There have been several prior case studies identifying the emergence of drug resistance in immunocompromised individuals (7, 14, 21, 38). Table S1 summarizes data for 43 patients over 26 publications. Of these, nine of the 10 who were reported to be treated with mAbs developed substitutions in the spike protein previously documented to confer resistance (7, 9, 12, 21). For our two patients treated with mAbs, *spike* K444N, conferring resistance to bebtelovimab, and *spike* E340D, conferring resistance to sotrovimab, emerged and became dominant by the next timepoint after treatment initiation. Bamlanivimab treatment has also been associated with development of *spike* E484K and E484Q antibody-resistant mutations in immunocompromised patients (12). Mutations in position 484 were commonly observed in studies of immunocompromised patients, but in our study, position 484 remained constant and contained the omicron-specific substitution E484A (11–13, 16, 18). Multiple further substitutions in spike have been reported in immunocompromised subjects but were not seen here (Table S1). Our findings thus are generally consistent with previous findings of the emergence of mAb resistance but emphasize the speed with which such changes can reach fixation (54, 55).

All patients in this study were treated with remdesivir, but we found no mutations annotated as conferring remdesivir resistance. In previous literature, 20 long-term infected patients were reported to be treated with remdesivir, and only one was reported to develop resistance mutations (6, 7, 9, 10, 12–17, 21, 24, 56–61) (Table S1). nsp12 E802D emerged as a remdesivir-resistant mutation in a patient with acquired B cell deficiency, suggesting that remdesivir drug resistance development is possible but not inevitable (21) and is less frequent than accumulation of resistance mutations to monoclonal antibodies. Possibly remdesivir resistance mutations confer a greater fitness cost to the virus, and therefore do not accumulate despite treatment (62).

We measured within-host evolution rates, quantified as the number of consensus substitutions per month, to be about two times higher than that observed with interhost background surveillance samples in three out of four of our patients. Over all four patients, a statistically greater mutational burden was documented in the *spike* coding region. The median estimated evolution rate across previous studies was 3.1 consensus substitutions per month. While some case studies reported no difference between intrahost and interhost evolution rates (16, 19, 20), other groups reported intrahost rates greater than the published rate for interhost variation (6, 7, 13, 15–17, 63) (Table S1). Accumulation of interhost substitutions in acute infections in otherwise healthy individuals appears to be minimal, although more data would be helpful to compare the evolution rates of acute and prolonged infections (46, 64, 65). These data suggest a high degree of variability within prolonged infection cases, although it is important to note that not all studies used the same approach to calculate evolution rates. A strength of our analysis is that we calculated the intrahost rate and interhost rate simultaneously using a representative sampling of contemporaneous genetically similar background isolates, facilitating direct and statistically robust comparison. These data highlight the potential for new viral evolution in immunocompromised patients.

Our study has several potential limitations. Our samples were collected as part of a retrospective study, as opposed to a prospective study with enrollment before infection. This results in sample collection that depends on the availability of previously banked specimens. In addition, our cohort size limited the statistical power of our analysis. The inability to reconstruct viral haplotypes is a current technical challenge in the field for within-host viral evolution studies, with the current 600-cycle Illumina kits not long enough to span most unique mutations for reconstruction. Additionally, this study represents a conservative estimate for evolutionary rates due to limitations of timepoints available for sampling. Some of the mutations most under selective pressure rise to dominance between sampling timepoints; therefore, it is possible that they dominated in fewer days than we estimated. Consequently, the estimates of fixation for iSNVs without intermediate timepoints collected represent a maximum amount of time. The patient’s iSNVs may have changed more rapidly than we could measure, given collection timepoints available. We did not have serum or blood cells available to monitor relevant immune responses. Interhost evolution rates for the BA.1 background appeared faster than other within-lineage Omicron rates (66). A possible explanation for this could be that the background included recombinant sublineages grouped in with BA.1 as labeled through GISAID’s lineage calls. Accumulation of iSNVs was analyzed using linear regression due to the number of timepoints available for each patient—with more timepoints collected a more thorough analysis could asssess whether linear curves best represent the data. Regressions that place zero-intercept before timepoint 0 suggest that the initial infection may have been seeded by a heterogeneous mixture. Finally, the study would have benefited from having a cohort of acute SARS-CoV-2 infections in otherwise healthy individuals with multiple timepoints to compare within-host evolution to immunocompromised individuals directly.

This study describes the evolutionary changes in immunocompromised, prolonged-infection individuals, including responses to drug treatment pressures. We identified efficient evolutionary escape from monoclonal antibody therapy, though not from remdesivir therapy. The emergence and onward transmission of mAb-resistant mutations could undermine the effectiveness of this element of our current therapeutic arsenal; our study provides an insight into outcomes in individual patients. Given a higher rate of SARS-CoV-2 evolution observed in a majority of our patients, our results underscore the importance of closely monitoring immunocompromised individuals as sources for new and concerning viral variants.

## MATERIALS AND METHODS

### Human subjects

Patients hospitalized at the Hospital of the University of Pennsylvania were enrolled following informed consent received under institutional review board-approved protocol #823392. Sample types included oropharyngeal and nasopharyngeal swabs or saliva, as previously described (67). Clinical metadata were manually abstracted from EPIC, the electronic medical record system used by the Hospital of the University of Pennsylvania. These abstracted data included the patients’ medical comorbidities, laboratory results, and medications administered. The laboratory data comprised lymphocyte and neutrophil counts recorded from the month prior to the date of the initial viral isolate to the date of the final isolate; both automated and manual cell counts were incorporated. Medication details provided the dates of administration spanning from the month prior to the initial viral isolate to the date of the final isolate, focusing on the following medication classes: anti-neoplastic agents, immunomodulators, and SARS-CoV-2-directed therapies. Medication administration was deemed continuous if doses were administered less than 5 days apart.

### Sequencing methods

The ARTIC POLAR protocol was used to obtain viral genomic sequences (doi: https://doi.org/10.1101/2020.04.25.061499). Sequences were obtained on Illumina NextSeq. Library preparation was performed as follows: a pre-reverse transcription reaction was performed with 5  µL of viral RNA, 0.5  µL of random hexamers at 50  µM (Thermo Fisher, N8080127), 0.5  µL of a 10  mM deoxynucleoside triphosphate (dNTP) Mix (Thermo Fisher, 18427013), and 1  µL of nuclease-free water heated for 5 minutes at 65°C and then incubated for 1 minute at 4°C. This followed a reverse transcription reaction using 6.5  µL of the above mixture combined with 0.5  µL of SuperScript III Reverse Transcriptase (Thermo Fisher, 18080085), 2  µL of 5× First-Strand Buffer (Thermo Fisher, 18080085), 0.5  µL of 0.1 M dithiothreitol (DTT) (Thermo Fisher, 18080085), and 0.5  µL of RNaseOut (Thermo Fisher, 18080051). This was incubated at 42°C for 50 minutes, at 70°C for 10 minutes, and then incubated at 4°C. The resulting amplicons from both primer sets for the sample were combined and then diluted to 0.25  ng/µL. For the cDNA amplification, artic-ncov2019 version 4.1 primers from IDT were used. The SARS-CoV-2 PCR was prepared using 2.5  µL of the previous mixture with 0.25  µL Q5 Hot Start DNA polymerase (NEB, M0493S), 0.5  µL of 10  mM dNTP Mix (NEB, N0447S), 5  µL of 5× Q5 reaction buffer (NEB, M0493S), and either 4.0  µL of primer set 1 or 3.98  µL of primer set 2 with nuclease-free water to achieve a 25  µL total volume. The PCR conditions were set to 98°C for 30  s (single cycle), followed by 25 cycles of 98°C for 15  s and 65°C for 5 minutes, and concluded at 4°C. The library was then prepared using the Nextera XT Library Preparation kit (Illumina, FC-131–1096) using IDT for Illumina DNA/RNA UD Indexes Set A, B, C, and D (Illumina, 20027213–20027216). The DNA content for each sample was gauged using the Quant-iT PicoGreen dsDNA quantitation kit (Invitrogen, P7589). After pooling samples in equal concentrations, the combined library’s quantity was determined using the Qubit1X dsDNA HS assay kit (Invitrogen, Q33230), and sequencing was performed on the Illumina NextSeq using a P1 2 × 300 chemistry.

### Sequence assembly

Sequence reads were first filtered to remove bases with a quality below Q20. These trimmed sequences were then aligned to the original Wuhan reference sequence (NC_045512.2) using the BWA aligner tool (v0.7.17). The Samtools package (v1.10) was used for filtering alignments. Variant positions were identified with the Bcftools package (v1.10.2–34), using PHRED scores of 20 or higher and variant read frequencies that constitute 50% or more of total reads. Variants were categorized using the Pangolin lineage software, specifically Pangolin version 4.1.3 coupled with pangolin-data 1.16. Point mutations were classified using a previously published bioinformatics pipeline (67). Key reagents are outlined in Table S5.

### Phylogenetic trees

Nextstrain’s augur tools (CLI v7.1.0) were used to generate representative subsamples of background surveillance data for each of the lineages detected in the five suspected prolonged-infection individuals. The earliest detection of the lineage, in the United States, with a complete, high-coverage genome was used as the root. Trees include all prolonged-infection samples and a 1:3 split of one USA sequence for every three tri-state sequences (Pennsylvania, New Jersey, or Delaware) to allow for a focused sampling in our region and representative samples from across the country. Trees were generated with 150–400 subsampled background sequences in addition to our prolonged infection samples. To generate trees, subsampled sequences were aligned with Nextclade v2.14.0, and maximum-likelihood phylogenetic trees with 1,000 bootstraps were generated using IQ-Tree v1.6.12^37^. Tree visualization was performed using iTOL v6.

### Root-to-tip analysis

Root-to-tip analysis was performed using the custom code to estimate evolution rates. Phylogenetic trees described above were analyzed used Ape (68) to compute the pairwise distance between the pairs of tips from the rooted phylogenetic trees. The root of the background sequences was selected as the earliest high-quality detection of the patient’s lineage in the United States, and the root for the prolonged-infection patient was the earliest sequenced timepoint collected. For each patient, the background and patient linear regressions are computed as follows:


$$distance=\;\beta_{0}+\;\beta_{1}*\;date+\;\varepsilon ,$$

where $\beta_{0}$ represents the intercept, $\beta_{1}$ represents the coefficient for date, and ε represents the error term. The 95% CI was calculated after estimating the standard error (SE) and the t-value using df=n−2 . These values were used to calculate the margin of error (MOE), where $MOE=t_{value}xSE$. The 95% CI for each coefficient β is estimated as Lower limit of C.I. = β−MOE and Upper  limit  of  C.I. =  β +MOE.

### iSNV statistical analysis

Major variants were called using bcftools, and iSNVs were called with bbtools (69, 70). Reads were filtered for a minimum mapping score of 40 from BWA and a quality score of 30. Patients were considered for analysis if they had two or more timepoints with two or more high-quality replicates per timepoint. High-quality replicates are defined as having 98% coverage with >200 × mean coverage, although 64 of our 71 samples had >1,000 × mean coverage. Regions with no coverage (containing base calls “Ns”) were excluded from any analysis. Thresholds for iSNVs were assessed at cutoffs for 0.01, 0.03, 0.05, 0.1, 0.15, 0.2, and 0.25. A cutoff of 0.03 with a required detection in two or more replicates was used to call subsequent iSNVs. All insertions, deletions, and substitutions (synonymous and nonsynonymous) that met these criteria were included. Coding region locations and lengths were used as described in NC_045512.2 (71). To determine if any coding regions had more or fewer than expected iSNVs, a linear regression was computed as defined by $n\;=\;\beta_{0}+\;\beta_{1}\;*\;length+\;\varepsilon$ , where n represents the n number of predicted unique iSNVs given the coding region length, $\beta_{0}$ represents the y-intercept, $\beta_{1}$ represents the coefficient associated with the coding region length, and ε is the error term capturing the variability in mutations not explained by coding region length. Adjusted *P*-values were obtained after calculating the standardized residuals (SR), where $SR=\frac{residuals}{standarddeviationofresiduals}$. Using the t-distribution, the two-tailed test’ s *P*-value for the SR was computed and then corrected for by using the Benjamini–Hochberg procedure to control for the false discovery rate. To estimate the rates of change in iSNVs over time, linear regression was used as described by $p=\;\beta_{0}+\;\beta_{1}\;*\;date+\;\varepsilon$, where $\beta_{0}$ represents the y-intercept, $\beta_{1}$ represents the coefficient associated with the coding region length, ε is the error term capturing the variability in mutations not explained by the coding region length, and p represents the proportion of reads containing n number of predicted unique iSNVs given the coding region length.

## Data Availability

Consensus sequences can be accessed on GISAID and GenBank with accessions listed in Table S2. Isolates used to generate phylogenetic trees can be accessed through their respective GISAID EPI_SET IDs. Patient 486 background isolates EPI_SET_231026xg (doi: 10.55876/gis8.231026xg). Patient 637 background isolates EPI_SET_231026tg (doi: 10.55876/gis8.231026tg). Patient 640 background isolates EPI_SET_231026pw (doi: 10.55876/gis8.231026pw). Patient 641 background isolates EPI_SET_231026yv doi: 10.55876/gis8.231026yv). Patient 663 background isolates EPI_SET_231026um (doi: 10.55876/gis8.231026um). Variant calls can be accessed in Table S6. Raw data can be accessed at BioProject PRJNA1011815. Computer code used can be accessed at https://github.com/andrewdmarques/Prolonged-Infection-Analysis

## SUPPLEMENTAL MATERIAL

Figure S1 mbio.00110-24-s0001.tif Deviation of iSNV proportion by replicate.

Figure S2 mbio.00110-24-s0002.eps Patient-specific testing results and medical records for symptom onset.

Figure S3 mbio.00110-24-s0003.eps Patient-specific phylogenetic trees containing lineage-specific background.

Figure S4 mbio.00110-24-s0004.eps iSNV by day.

Figure S5 mbio.00110-24-s0005.tif Linear regression for iSNV viral population rates of change.

Tables S1 to S6 mbio.00110-24-s0006.xlsx Tables S1 to S6.

ASM does not own the copyrights to Supplemental Material that may be linked to, or accessed through, an article. The authors have granted ASM a non-exclusive, world-wide license to publish the Supplemental Material files. Please contact the corresponding author directly for reuse.
